# Creating Wikipedia articles on health and technology topics can empower the writers and benefit the community

**DOI:** 10.1186/s12909-022-03389-5

**Published:** 2022-05-16

**Authors:** Khen Moscovici, Gal Ben Arie, Ilan Shelef, Adina Kalet

**Affiliations:** 1 Division of Diagnostic and Interventional Imaging, Soroka University Medical Center, Beer-Sheva, Israel; 2 Robert D. and Patricia E. Kern Institute for the Transformation of Medical Education, Medical College of Wisconsin, 8701 W. Watertown Plank Road, Wauwatosa, WI 53226 USA

## Abstract

**Background:**

Publicly accessible information regarding imaging procedures is lacking, especially in non-English languages. Biomedical engineering students do not generally have opportunities to practice conveying scientific knowledge to the public.

**Methods:**

As part of a Techniques and Clinical Usage of Medical Imaging Devices course, for extra credit, several biomedical engineering students choose to create and edit Wikipedia articles in the local language (Hebrew). The goal of this activity was to serve the local community, while improving students’ abilities and self-perception in reading and reporting scientific knowledge. Following task completion, individual interviews were conducted with the students to assess the impact of the task on student personal development, sense of meaning and their view of their role in educating the public.

**Results:**

Most students considered the task meaningful and impactful on society. Additional academic credit was not perceived as the most important incentive for participating.

**Conclusions:**

Medical and other professional schools should seek to include tasks such as writing Wikipedia articles in their curricula. Educational assignments that integrate academic work, student identity development and direct community benefit can have a long-term beneficial impact on learners and society.

**Supplementary Information:**

The online version contains supplementary material available at 10.1186/s12909-022-03389-5.

## Background

Founded in the seventeenth century, scientific publishing attempted to promote the spread of knowledge by bridging the academy-public knowledge gap [1]. Despite the fact that today over 25,000 scientific journals exist, and 1.8 million articles are published yearly [1], the vast majority of scientific knowledge is still not easily accessible to the public because journal subscriptions are costly [1] and most are written in a format and style not easily comprehensible to the average reader. Thus, regardless of the attempt to bring knowledge to the public through scientific publishing little seems to have changed [2].

Nowadays, members of the public generally find answers to questions about medicine and healthcare fields on the internet [3]. In this respect, the medicine and healthcare fields do not differ from other disciplines [4, 5]. Patients actively search for medical information online, mainly relying on search engines such as Google, Yahoo and MSN [6, 7], which are not managed by medical professionals [8]. A number of studies have demonstrated that media sources commonly used by the public, such as those providing news about medical conditions, can be misleading or incomplete [7, 9]. The exponential growth of knowledge, together with its limited dissemination to the public, has led universities to explore alternative methods of spreading knowledge, outside of scientific publishing. Some of these methods include encouraging op-ed writing by researchers, and using Blogs, Twitter, and other publishing forms aimed at the general public [8, 10].

Multidisciplinary training is needed to ensure that professionals from all relevant disciplines are able to provide accessible high quality professional knowledge to the public [11, 12]. The rise in use of medical imaging is among the most important technical advances in medicine in recent years [13]. We sought to engage bioengineering students and enable them to bridge the academy-public knowledge gap in medical imaging. Current medical trends dictate that most people undergo several imaging exams in their lifetime. Indeed, chest x-rays, ultrasound, computed tomography (CT) and Magnetic Resonance Imaging (MRI) have become extremely common in everyday medical practice [14–16] while there has not been a commensurate expansion of reliable information accessible to patients. Wikipedia, a free online rigorously peer reviewed encyclopedia is among the top websites in the health-related topics for the public [6, 17]. A number of studies reported that 90% of engineering students and over 50% of physicians, especially junior physicians, use Wikipedia frequently for academic and professional purposes [18, 19]. However, Wikipedia’s information is not readily available in non-English languages [20].

As in the proverb of the blind men and the elephant, patients, physicians, and biomedical engineers perceive medical imaging (the elephant in this case) differently, with distinct scopes of knowledge, understanding and expectations. There is a need to both prepare biomedical engineers to collaborate and communicate scientific knowledge across professions and with the public and to build capacity to provide much needed information to Hebrew-only reading populations (there are approximately 9 million Hebrew speakers worldwide). In this curricular innovation, we evaluated the impact of an assignment to write Wikipedia articles in the medical imaging field on students’ ability to access and interpret the relevant scientific knowledge and their motivation to contribute to the public good.

## Methods

### Curriculum

‘Techniques and Clinical Usage of Medical Imaging Devices’ is an elective course held in our institute for 3rd and 4th year biomedical engineering students (4 years degree). The course (taught by a clinical radiologist, GBA) aims to introduce the clinical aspects of medical imaging to future engineers and to encourage a link between clinicians and engineers. Among the learning objectives of the course is to prepare students to disseminate scientific knowledge to the general Hebrew speaking public (the full course curriculum presented in Additional file: 1 Appendix 1). Due to the nature of the task and anonymity of the interviews, we were unable to assess and include any demographic data about the students who participated in this project.

### Intervention

Students (during 2019–2020), working individually or in pairs, could elect to write or edit a Wikipedia article in the field of medical imaging for extra course credit. They were required to review the current literature and to reference all the available knowledge indexed in PUBMED. Wikipedia articles are intended for use by the public, and thus, students were expected to rewrite current data in a style and literacy level that would be accessible to the general public. Students were permitted to select any topic in the scope of biomedical engineering, and to choose one of two options: writing a new Hebrew Wikipedia article (a minimum of 300 words, or if the article existed in a different language the addition of 50 new words and at least 2 new scientific references was required) or editing an existing Hebrew Wikipedia article (while adding at least 300 new words and at least 8 scientific references). When contacted, the local Wikipedia Non Governmental Organization recommended writing new articles as opposed to editing existing ones, as the best means to impact society as reflected in the number of views. Course faculty approved the topics prior to task commencement, and guided the students, throughout the task, on selection of high quality literature and clarity and technical quality of the writing. Upon completing the task, each student received 5 bonus points to the final grade (of 100 points) in a binary manner (all or none).

### Student assessment

Wikipedia articles written by the students were assessed for quality and accuracy of the scientific writing by a diagnostic radiology specialist (GBA) after the material was uploaded to the Wikipedia draft space, but prior to online publishing and peer review.

### Evaluation

To evaluate students’ motivation and the educational impact of the task, we distributed questionnaires to those who chose not to perform the task and interviewed the students who completed the task. Questionnaires for the non-participating students contained two open questions. Students were asked to cite the main reason for not undertaking the task and how they perceived the task. To avoid any pressure to perform the task, the questionnaire was delivered only after the course ended. An individual independent of the course faculty interviewed all students who completed the task. The interviews were semi-structured (see Additional file: 1 Appendix 2) and addressed three main topics: 1) Perception of societal and personal gaps in medical knowledge 2) Wikipedia as a platform to address those knowledge gaps 3) personal competence during academic studies, and 4) the experience of the task. Data collected from the questionnaires and interviews were thematically analyzed using Braun and Clarke’s framework [21, 22]. The text from the questionnaires was compiled and interviews were audio recorded and then transcribed by the independent interviewer who removed all personally identifying information. The independent reviewer has summarized points from all the interviews (3000 words total approximately in length) and submitted it to the authors, to prevent any personal bias of recognizing any detail or voice of students.

## Results

Of the 28 students enrolled in the course, 13 participated in the described task and wrote 7 new Hebrew Wikipedia articles in the medical imaging field. None of the students decided to edit an existing Hebrew Wikipedia article. All but one student opted to complete the assignment collaboratively in pairs.

All the articles were approved for publishing by the local community peer reviewers, and as of November 12th, 2021 (fourteen months from the end of the course), these seven articles were viewed 17,154 times.

All students who performed the task were in the second half of the undergrad degree. Given the small number of participants and the choice to maintain confidentiality we did not collect individual descriptive characteristics. Most students who participated did not have any prior Wikipedia editing experience, except one student. All students expressed a high level of English and Hebrew language literacy and did not expect that reading, interpreting, or writing about medical and scientific materials would pose insurmountable difficulties in preparing the task. Nor were they concerned about navigating the Wikipedia technical environment.

### Motivation to undertake the task: contributing to society

Students cited reasons for undertaking the task including; a sense of meaning in performing an academic task that is accessible to all; an opportunity to contribute to society; and a personal desire to seek professional medical knowledge. Interestingly, both students who did and those who did not complete the task stated that the bonus grading (5 points of 100) was perceived as inadequate for the time they estimated the task would demand.

Most students (9 of 15) who did not undertake the task cited their apprehensions regarding their workload during the semester. Nonetheless even those students who did not participate perceived the task as meaningful.

### Challenges in completing the task: topic selection

Students mentioned that the scientific reading was the most time-consuming part of the task and it added significantly to their workload, as they expected. However, they encountered little to no technical difficulties in handling the Wikipedia website. According to the students their main challenge was to select content important to both lay people and medical professionals. Only a minority of the students (2 of the 13) revealed that a personal medical experience was the main consideration in choosing a specific topic.

### Student satisfaction and concerns

Most (8 of 13) of the students who performed the task were satisfied with the results. Satisfaction was expressed several times during the interviews in phrases such as - *“This was one of the only practical assignments in my studies that could make an impact”*. Those who were not satisfied claimed that they were limited in writing about medical topics due to their training in engineering. Further, some expressed concern that the encyclopedic writing style requirements of Wikipedia does not fully convey the intended message to the public. These respondents felt that popular health media, such as health sections in news websites, could also contribute to society’s knowledge. Students stated that the task differed from other tasks in their academic studies in many aspects, including: *the* c*ontribution to society, the uniqueness of the research experience, and writing about medical knowledge for the general public.*

When asked to elaborate on the above mentioned aspects, students defined ‘contribution to society’ as an action which reduces medical knowledge gaps between the general public and professionals. ‘Unique research and writing about medical topics’ were described as the single task in their training, which required them to go beyond reading only about technical engineering topics, and they appreciated the challenge and value of learning to translate and simplify these complex ideas to be understandable by the general public. Students stated that in contrast to other academic tasks that were not published and kept inside the academy ‘walls’, this task was published almost immediately to the general public as a Wikipedia article, easily accessible from every search engine. Completing a task that pushes through ‘the boundaries of the classroom’ contributed to the students’ feeling that they can have an impact on society. Accordingly, most stated that they did not feel that their skills and abilities in completing tasks under pressure or in summarizing and editing information improved.

## Discussion

Students of biomedical engineering were offered, and half participated in, an academic assignment with an opportunity to practice the communication of scientific knowledge to the public. This task was unique in that it required students to interact with the public in nonacademic domains. Most of the students who participated in the course, including those who did choose to do the assignment, felt that it was meaningful and original. While it is possible that students were initially motivated simply by the opportunity to receive additional credit in in this competitive degree program, in the end all students acknowledged that the bonus credit was modest for the effort required and recognized that the real value of the task was the opportunity to contribute to learn how to interpret complex scientific ideas for the lay public. These are skills that are likely to serve them well throughout their careers.

### Knowledge dispersion in the information age

Patients and doctors consume more and more information from nonacademic sources [6, 7], such as websites and social networks. Much of this information is not reliable nor peer reviewed. We aimed to engage students in transforming academic knowledge into an accessible nonacademic source that can increase the health literacy of the general public. Limitations in delivering knowledge using this method include steering students toward selecting appropriate topics for the task and advertising the availability of the information to the person in need of it.

## Conclusions

### Generating public knowledge with an academic course

Every non-English language speaking community needs better access to medical knowledge. Designing educational activities across the health professions including medical students, nursing, pharmacy students among others, especially in language groups with relatively small populations can ensure such populations have broad access to information. Our very limited experience piloting this novel approach to involving students in generating high quality public information suggests that it could have great value if implemented as a mandatory task.

As next steps we hope to study the impact of this educational activity on; the development of a student’s skills, professional identity and involvement in multidisciplinary collaborations and direct impact on improving the public’s access to scientific information. Practices and conclusions from this experience with biomedical engineering students can be easily applied to other academic faculties or departments improving on levels of general and health literacy levels in the targeted communities.

## Supplementary Information

**Additional file 1.**

## Data Availability

The datasets generated and/or analyzed during the current study are not publicly available due to its nature, it is recordings in Hebrew of meetings and follow up with students, that do not contain any personal information but are personal in nature. The analysis and transcripts are available from the corresponding author on reasonable request.
