# Supplementary material for: Creating Wikipedia articles on health and technology topics can empower the writers and benefit the community
Source: BMC Med Educ. 2022 May 16;22:373. doi: 10.1186/s12909-022-03389-5 (PMC9109402; doi:10.1186/s12909-022-03389-5)
Supplement: Supplementary file 1 — Additional file 1. [file 12909_2022_3389_MOESM1_ESM.docx]

Appendix 1 – Course Curriculum

The course consists of 10 sessions (each is 2 academic hours long). The course description in the syllabus is as follows:

This course is designed to provide students with an understanding that integrates engineering, biological and medical aspects of imaging devices, and of how technology and engineering enables and advances imaging and medical care. The devices to be discussed are: standard X-ray, ultrasound, Computerized Tomography (CT), Magnetic Resonance Imaging (MRI), catheterization, nuclear medicine and computing systems for maintaining and managing imaging systems.

The subjects and activities for the course include:

- X-ray
- Computerized Tomography imaging
- Ultrasound imaging
- Magnetic Resonance Imaging
- Cardiology CT, MRI
- Medical imaging and artificial intelligence
- Advanced brain imaging (including stents therapy)
- Nuclear medicine
- Imaging of the various body systems
- Computing systems for maintaining and managing imaging systems
- Tour in the imaging department

Appendix 2 – Interview statements

Students were asked whether they agree with the following statements:

- Gaps in knowledge and medical information exist (their opinion before undertaking the task)
  - There are significant gaps in medical knowledge in society.
  - Professional knowledge should be in the hands of professionals, and not the public.
  - It is easier for an English speaker to acquire knowledge than for other language speakers.
  - It is important for me to make knowledge accessible to the public in the context of my academic studies.
  - I believe that I can contribute to society during my studies (and not just after).
  - I can work as part of a team.
  - I believe that I can write clearly and to a wide audience.
  - I have the ability to read and understand medical information.
  - I believe I can easily do a literature review.
  - I believe that the public can make educated guesses.
- Selecting the medical topic and views on Wikipedia platform
  - Wikipedia as a useful tool (thoughts before and after completing the task)
    - I think Wikipedia is a reliable source of information.
    - I believe I understand what information people search on Wikipedia.
    - I believe that I can easily write an entry on Wikipedia.
- Personal competence, course experience, thoughts on the task
  - How was the communication with the course staff? The ongoing work on the task? (open-ended)
  - Now that the task is over, I feel that my abilities to read and understand scientific information have changed.
  - The task has improved some of my skills.
  - Now that I have completed the task, I feel that my ability to disseminate knowledge to the general public has changed.
  - Now that I have completed the task, I feel that the content I created will contribute to society.
  - Now that I have completed the task, I believe that someone I know personally will use the content I created.
  - Now that I have completed the task, I appreciate people who engage in making scientific knowledge accessible to the general public (e.g. health correspondents) more.
  - Now that I have completed the task, I appreciate the skill / complexity of making scientific knowledge accessible to the general public.
  - Now that I have completed the task, I feel that students can make a change in society (and not just after their studies).
  - Do you think completing the task is worth the bonus points given? (open-ended)
  - In comparison to other courses in my degree, I feel that the course is **different** / **similar** in terms of the learning experience.
  - The course did not affect my perception of academic studies.

Appendix 3

The list of Wikipedia articles and view count for each article created for this project can be accessed with this link:

https://pageviews.toolforge.org/?project=he.wikipedia.org&platform=all-access&agent=user&redirects=1&start=2019-12-10&end=2021-11-11&pages=%D7%92%D7%A0%D7%98%D7%A8%D7%99_(%D7%A8%D7%A4%D7%95%D7%90%D7%94)|%D7%98%D7%95%D7%9E%D7%95%D7%92%D7%A8%D7%A4%D7%99%D7%94_%D7%9E%D7%9E%D7%95%D7%97%D7%A9%D7%91%D7%AA_%D7%A9%D7%9C_%D7%94%D7%A8%D7%90%D7%A9_(%D7%A1%D7%99-%D7%98%D7%99)|%D7%93%D7%99%D7%95%D7%95%D7%97_%D7%95%D7%9E%D7%A2%D7%A8%D7%9B%D7%AA_%D7%A0%D7%AA%D7%95%D7%A0%D7%99%D7%9D_%D7%A2%D7%9C_%D7%93%D7%99%D7%9E%D7%95%D7%AA_%D7%9B%D7%91%D7%93|%D7%A7%D7%95%D7%9C%D7%95%D7%A0%D7%95%D7%A1%D7%A7%D7%95%D7%A4%D7%99%D7%94_%D7%95%D7%99%D7%A8%D7%98%D7%95%D7%90%D7%9C%D7%99%D7%AA|%D7%90%D7%95%D7%9C%D7%98%D7%A8%D7%90%D7%A1%D7%90%D7%95%D7%A0%D7%93_%D7%90%D7%A0%D7%93%D7%95%D7%A1%D7%A7%D7%95%D7%A4%D7%99|%D7%91%D7%93%D7%99%D7%A7%D7%AA_%D7%90%D7%95%D7%9C%D7%98%D7%A8%D7%94_%D7%A1%D7%90%D7%95%D7%A0%D7%93_%D7%A8%D7%A4%D7%95%D7%90%D7%99%D7%AA|%D7%97%D7%95%D7%9E%D7%A8_%D7%A0%D7%99%D7%92%D7%95%D7%93_%D7%A8%D7%93%D7%99%D7%95%D7%9C%D7%95%D7%92%D7%99
